# Retrospective study of elderly patients with advanced ovarian cancer who did not undergo surgery

**DOI:** 10.1093/oncolo/oyaf290

**Published:** 2025-09-18

**Authors:** Hui Qu, QianXue Wei, Wen Gu, Zhenhua Du, Xiuqin Li, Yu Xia

**Affiliations:** Department of Obstetrics and Gynecology, Shengjing Hospital of China Medical University, Shenyang, 110004, China; Department of Obstetrics and Gynecology, Shengjing Hospital of China Medical University, Shenyang, 110004, China; Department of Oncology, Shengjing Hospital of China Medical University, Shenyang, 110004, China; Department of Obstetrics and Gynecology, Shengjing Hospital of China Medical University, Shenyang, 110004, China; Department of Obstetrics and Gynecology, Shengjing Hospital of China Medical University, Shenyang, 110004, China; Department of Obstetrics and Gynecology, Shengjing Hospital of China Medical University, Shenyang, 110004, China

**Keywords:** elderly, advanced ovarian cancer, comorbidity, chemotherapy, maintenance therapy, overall survival

## Abstract

**Objective:**

To evaluate the clinical outcomes of elderly patients with advanced ovarian cancer who did not undergo surgery and received chemotherapy with or without maintenance therapy.

**Methods:**

We retrospectively analyzed the clinical data of 15 patients with advanced high-grade serous ovarian cancer who were treated at our hospital between 2018 and 2023. These patients either had multiple comorbidities or refused surgery. Data collected included patient demographics, treatment regimens, chemotherapy cycles, clinical response, progression-free survival (PFS), and overall survival (OS).

**Results:**

The median age of the patients was 73 years (range, 50-86 years). Fourteen patients received platinum-based chemotherapy combined with paclitaxel or liposomal doxorubicin, with or without bevacizumab, for 3 to 6 cycles. Twelve patients who achieved disease control received PARP inhibitor maintenance therapy. The overall response rate (ORR) was 80.0%, or 12/15 patients achieved partial response (PR); nobody achieved complete response. The disease control rate (DCR) was 100%. The median PFS1 was 19.0 months (95% CI, 11.85-26.15), and the median PFS2 was 10 months. The 3-year OS rate was 65.2%, with a median OS of 57.0 months (95% CI, 13.00-100.99).

**Conclusions:**

Chemotherapy with or without bevacizumab, followed by PARP inhibitor maintenance therapy, is a viable alternative for elderly or surgically ineligible patients with advanced ovarian cancer. The findings of this study should be considered exploratory and require validation through large-scale studies.

Implications for Practice This study offers a feasible non-surgical treatment strategy and provides specific clinical guidance for patients with advanced high-grade serous ovarian cancer who are ineligible for tumor debulking surgery due to advanced age, severe comorbidities, or personal preference. In clinical practice, a platinum-based doublet chemotherapy regimen (paclitaxel/liposomal doxorubicin plus platinum) may be considered for this specific population, with the optional addition of bevacizumab depending on patient tolerance. Patients who achieve disease control (partial response or stable disease) can then receive PARP inhibitor maintenance therapy. This approach is particularly suitable for elderly patients with multiple chronic conditions and poor physical function. It is recommended that an individualized treatment strategy be developed through multidisciplinary team collaboration, which should comprehensively assess the patient’s general condition, complication risks, treatment tolerance, and personal preferences to optimize the benefit-risk balance. It should be noted that this study does not replace current guideline-recommended standard treatments but merely offers an alternative therapeutic option for patients unable to undergo surgery. Furthermore, we emphasize the necessity of conducting randomized controlled trials in the future.

## Introduction

Ovarian cancer primarily affects elderly women, with most cases occurring in those aged 65 and older.^1^ Research has shown that advanced age is a significant independent risk factor for reduced survival rates in elderly ovarian cancer patients.^1–3^ Historically, clinical practice has demonstrated that satisfactory tumor cytoreduction (R0/R1 resection) is crucial for improving outcomes in patients with stage III and IV ovarian cancer. Over the past decade, surgical techniques for ovarian cancer have advanced significantly. To achieve R0 resection, surgeons often remove parts of the intestines, peritoneum, liver, spleen, and cardiophrenic lymph nodes. These procedures are extensive, lengthy, and highly invasive, resulting in significant surgical trauma and slow postoperative recovery for patients. However, most clinical trials have strict inclusion criteria, typically limiting participants to those under 70 years of age and excluding those with comorbidities. As the global population ages, the number of elderly individuals with multiple chronic diseases is increasing. Consequently, the number of patients who cannot undergo surgery due to advanced age or comorbidities is expected to rise. Does this mean that patients who do not undergo surgery are destined to have a poor prognosis? To explore this question, we conducted a retrospective analysis of the clinical outcomes of 15 patients who declined surgery due to comorbidities or advanced age.

## Materials and methods

### Study design and participants

This study is a retrospective clinical investigation. The inclusion criteria encompassed patients diagnosed with advanced epithelial ovarian cancer at our center (Department of Gynecology, Shengjing Hospital of China Medical University) from 2018 to 2023. Specifically, those who were either unable to undergo ovarian cancer tumor debulking surgery or subjectively refused it, and who had a survival duration of at least 6 months, were included. Comprehensive clinical data were collected for the enrolled patients, covering basic characteristics, treatment modalities, chemotherapy courses, treatment responses, progression-free survival (PFS), overall survival (OS), and other relevant parameters. As a retrospective study, this research has secured approval from the ethics committee for a waiver of informed consent.

### Treatment methods

None of the patients underwent tumor cytoreductive surgery due to comorbidities or personal preferences. Instead, they received systemic intravenous chemotherapy for 3 to 6 cycles. In accordance with the guidelines, the first-line chemotherapy regimens included paclitaxel/liposomal doxorubicin combined with platinum-based regimens, or 2 sequential regimens based on the patient’s tumor assessment. Some patients also received bevacizumab in combination with their chemotherapy. Following chemotherapy, all but 3 patients received PARP inhibitor maintenance therapy. The exceptions were 2 patients who did not have access to PARP inhibitors at the time and one patient who was unable to afford the treatment. The basic clinical characteristics of the patients are summarized in Table 1. Detailed treatment information for all patients included in the study (designated as P1 to P15) is presented in Table 2.

**Table 1.** Baseline patient and disease characteristics.

	*n* (%)
**FIGO stage**	
**III**	7 (46.7)
**IV**	8 (53.3)
**ECOG performance status**	
**0**	0
**1**	8 (53.3)
**2**	7 (46.7)
**Histology type**	
**Serous**	15 (100%)
**Others**	0
**Number of organs with metastases**	
**≤2**	0
**>2**	15 (100%)
**PARPi maintenance at initial treatment**	
**Yes**	12 (80.0%)
**No**	3 (20.0%)

**Table 2.** Treatment and prognosis of 15 patients.

Patient number	Age (years)	Stage	Comorbidities and reasons for not undergoing surgery	Therapy regimen	Reasons for not completing chemotherapy	Efficacy evaluation	Maintenance therapy	PFS1 (months)	PFS2 (months)	OS (months)
P1	76	III	Pulmonary embolism; Lower limb thrombosis; patient refused surgery	TC*4; Liposomal Doxorubicin + Oxaliplatin4	Not applicable	PR	Niraparib	12	5	34
P2	51	IVB	Severe coronary stenosis; hypertension stage II; patient refused surgery	Docetaxel + Cisplatin*6	Not applicable	PR	Fluzoparib	15	7	26+
P3	50	IVB	Hypertension stage II; patient refused surgery due to financial reasons	TC*6 (Refusal of sequential chemotherapy)	Not applicable	PR	None	9	18+	27+
P4	75	IVB	Facial nerve paralysis; hypertension stage II; patient refused surgery	TC + Bevacizumab (intraperitoneal infusion)*3	Severe neutropenia with septic shock; patient refused further chemotherapy	SD	Fluzoparib	15	6	25
P5	86	III	Rheumatoid arthritis; patient refused surgery due to advanced age	TC*3	Herpes zoster with severe postherpetic neuralgia	SD	Fluzoparib	25+	Not applicable	25+
P6	56	IVB	Metastasis in the upper abdomen involving the pancreas head and duodenum; patient refused surgery	TC + Bevacizumab*3; Liposomal Doxorubicin + Cisplatin + Bevacizumab*3	Not applicable	PR	Pamiparib	20	Not applicable	20
P7	73	III	History of thyroid cancer; hypertension stage II; patient refused surgery	TC*3; Paclitaxel + Oxaliplatin*3 (Bevacizumab not used due to pulmonary embolism)	Not applicable	PR	Fluzoparib	19	5+	24+
P8	69	III	Hypertension stage II; patient refused surgery	TC*3; Paclitaxel + Oxaliplatin + Bevacizumab*3	Not applicable	PR	Fluzoparib	20+	Not applicable	20+
P9	65	IVB	Patient refused surgery	TC + Bevacizumab*4	Not applicable	PR	Fluzoparib	14	5+	19+
P10	76	III	Patient refused surgery due to advanced age	TC*2; Liposomal Doxorubicin + Oxaliplatin1	Patient stopped treatment voluntarily	PR	None	18	10	57
P11	76	IVB	Patient refused surgery due to advanced age	TC*6	Not applicable	PR	Niraparib	44	16+	60+
P12	61	IVB	Right tibial fracture after 3 cycles of neoadjuvant chemotherapy; patient refused surgery	Liposomal Doxorubicin + Carboplatin*6	Not applicable	PR	None	66	14+	80+
P13	73	III	Splenic metastasis; hypertension stage II; patient’s family refused surgery	TC*3	Patient unable to tolerate chemotherapy side effects	SD	Fluzoparib	13+	Not applicable	13+
P14	81	III	Hypertension stage II; history of liver cancer; hepatitis B and liver cirrhosis; patient refused surgery and chemotherapy	Bevacizumab immunotherapy	Not applicable	SD	Fluzoparib + immunotherapy	11	3	16+
P15	71	IVB	Patient refused surgery and chemotherapy due to advanced age	TC*6	Not applicable	PR	Niraparib	39+	Not applicable	39+

Abbreviations: NA, not applicable; OS, overall survival; PFS1, progression-free survival 1; PFS2, progression-free survival 2; TC, paclitaxel + carboplatin; +, patient has not experienced progression or death event.

### Clinical efficacy evaluation standards and main study endpoints

#### Clinical efficacy evaluation

At the conclusion of initial chemotherapy, clinical efficacy was assessed according to the RECIST (Response Evaluation Criteria in Solid Tumors) version 1.1 criteria. The criteria encompass 4 categories: complete response (CR), partial response (PR), stable disease (SD), and progressive disease (PD). Specifically, a CR is defined as the disappearance of all target lesions, with the short axis of any pathological lymph nodes (whether target or non-target) reduced to less than 10 mm. A PR indicates that the sum of the diameters of all target lesions has decreased by at least 30%. PD is characterized by an increase of at least 20% in the sum of the diameters of target lesions (Note: The appearance of one or more new lesions is also considered as disease progression). SD is defined as insufficient shrinkage to qualify for PR or insufficient enlargement to qualify for PD.

#### Primary endpoint

The primary endpoint of this study is PFS1, which is the time interval from the start of treatment to the first recurrence. Researchers evaluated the time from treatment initiation to tumor progression (in any aspect) in subjects according to the RECIST 1.1 criteria.

#### Secondary endpoints

The secondary endpoints include objective response rate (ORR), disease control rate (DCR), PFS2, and OS. PFS2 is defined as the time from the first radiological progression (RECIST 1.1) to the second radiological progression or death from any cause. Even if subsequent anti-tumor therapy (eg, second-line chemotherapy or targeted maintenance) is administered after initial progression, the PFS2 start date remains the date of first progression. The endpoint is the second radiological progression or death, whichever occurs first. The ORR refers to the proportion of patients whose tumor volume decreases to a predetermined value and can be maintained for the minimum required duration. It is calculated as the sum of the proportions of patients achieving CR and PR. The DCR is defined as the ratio of the sum of cases achieving either a response (PR + CR) or SD after treatment to the total number of evaluable cases.

### Safety

The safety profile of the treatment was evaluated by assessing adverse events that occurred during the patients’ treatment. These events were graded according to the National Cancer Institute’s Common Terminology Criteria for Adverse Events (CTCAE) to determine their occurrence and severity. The adverse reactions observed included both hematological toxicities (such as anemia, thrombocytopenia, neutropenia, and leukopenia) and non-hematological toxicities (such as elevated transaminase levels, fatigue, nausea, vomiting, and diarrhea).

### Statistical analysis

Statistical analysis was conducted using SPSS 25.0 software. Qualitative data were presented as percentages. The Kaplan–Meier method was employed to analyze PFS and OS, including median PFS values, PFS rates, median OS values, and OS rates. Graphical representations were generated using R version 4.2.2. The significance level (α) was set at 0.05.

## Results

### Patient characteristics

Among the 15 patients included in the study, the median age was 73 years, with a range from 50 to 86 years. All patients were diagnosed with high-grade serous epithelial ovarian cancer. At baseline, 8 patients (54%) had an ECOG Performance Status (PS) score of 1, while 7 patients (46%) had a score of 2. Each patient had at least 2 metastatic sites. The median follow-up duration was 25 months, ranging from 13 to 80 months. The detailed baseline disease characteristics of the patients are presented in Table 1.

### Treatment method

With the exception of patient P14, all other 14 patients received systemic intravenous chemotherapy with platinum-based plus paclitaxel/liposomal doxorubicin regimens, with or without the addition of intravenous bevacizumab. The standard treatment course consisted of 6 cycles. Among these patients, 5 (P1, P6, P7, P8, and P10) underwent sequential treatment with 2 first-line chemotherapy regimens. Additionally, 5 patients (P4, P5, P10, and P13) were unable to complete the full course of chemotherapy due to personal reasons or severe side effects encountered during treatment. Four patients (P4, P6, P8, and P9) received bevacizumab in combination with chemotherapy. Following chemotherapy, 12 patients who achieved disease control continued with PARP inhibitor maintenance therapy. Three patients (P3, P1, and P12) opted for observation after chemotherapy. Patient P14 initially declined intravenous chemotherapy and requested anti-angiogenic targeted therapy combined with immunotherapy. The comorbidities, reasons for not undergoing surgery, therapy regimens, reasons for incomplete treatment courses, and maintenance therapies for each patient are detailed in Table 2.

### Efficacy

**Figure 1.**
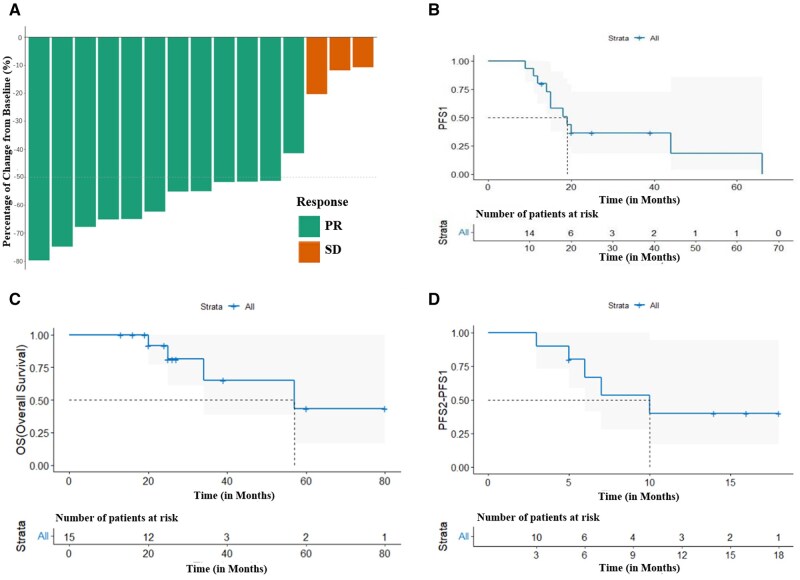
Treatment outcomes and survival analysis. (A) Best percentage change in tumor lesion size from baseline. A negative value indicates that the tumor size was smaller at follow-up compared to baseline. (B) Kaplan–Meier estimates of progression-free survival (PFS1). (C) Kaplan–Meier estimates of overall survival (OS). (D) Kaplan–Meier estimates of progression-free survival (PFS2).

All 15 patients were included in the short-term efficacy evaluation. According to the RECIST 1.1 criteria, nobody achieved CR. Twelve out of 15 patients (80.0%) achieved PR, while 3 out of 15 patients (20.0%) had SD (see Table 2). The ORR was 80.0%, or 12 of 15 patients achieved PR. The DCR was 100% (15/15). The changes in target lesions compared to baseline are illustrated in Figure 1.

The follow-up cutoff date was November 2024. For the 15 patients assessed by the investigators, the median PFS1 was 19.0 months (95% CI = 11.85-26.15), with a range from 9 to 66 months. The 1-year PFS rate was 80.0%, the 2-year PFS rate was 36.4%, and the 3-year PFS rate was 36.4% (see Figure 1). The median OS for the 15 patients was 57.0 months (95% CI = 13.00-100.99) (range 13-80 months). The 1-year OS rate was 100.0%, the 2-year OS rate was 91.7%, and the 3-year OS rate was 65.2% (see Figure 1). Among the 10 patients who received subsequent treatment after the first recurrence, the median PFS2 was 10 months (see Figure 1).

**Figure 2.**
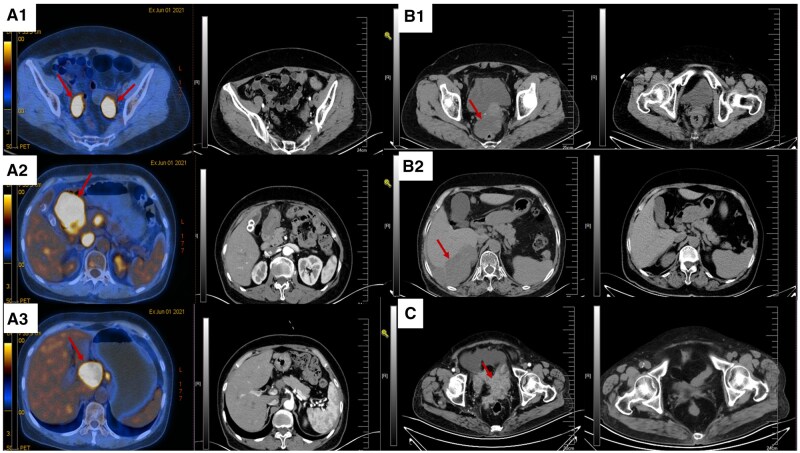
Comparison of CT images before and after treatment. (A) 1-3. Patient P6. (B) 1-2. Patient P9. (C) Patient P10. The lesion indicated by the arrow corresponds to the baseline lesion prior to treatment.

The PFS1, PFS2, and OS results for each patient are detailed in Table 2. In this study, we conducted a comparative analysis of CT images before and after treatment for selected patients. The comparative analysis of CT images for these patients is presented in Figure 2.

### Safety

All 15 patients were included in the safety analysis (see Table S1). The most common hematological toxicities included leukopenia (93.3%), with a grade 1-2 incidence of 73.3%; neutropenia (93.3%), with a grade 1-2 incidence of 53.3%; and thrombocytopenia (73.3%), all of which were grade 1-2. The primary non-hematological toxicities were fatigue (100%), vomiting (100%), and nausea (100%), all of which were grade 1-2.

## Discussion

After decades of clinical practice and exploration, primary debulking surgery has long been the preferred treatment for patients with stage III and IV ovarian cancer. Achieving satisfactory tumor cytoreduction (R0/R1) is a crucial determinant of patient survival. To meet the R0/R1 surgical standards, it is often necessary to resect parts of the bowel, peritoneum, liver, spleen, and other organs. This can subject patients to significant surgical trauma and prolonged postoperative recovery. In cases where advanced ovarian cancer patients have extensive tumor involvement and satisfactory debulking cannot be achieved, neoadjuvant chemotherapy followed by interval debulking surgery serves as a viable alternative.^4–8^

However, patients in those randomized controlled trials are typically selected based on strict inclusion criteria, often being under the age of 70 and free from comorbidities. With the increasing aging population and extended human lifespan, approximately 70% of elderly patients have at least one chronic disease, and a significant proportion may have 2 to 3 comorbidities. The proportion of elderly patients with ovarian cancer is expected to continue rising, and these patients face significantly higher perioperative risks, postoperative severe complication rates, and mortality rates.^4–6^ In the real world, how should clinicians make informed decisions for such patients?

In this study, the median age of the 15 patients was 73 years. Seven patients (46%) had an ECOG score of 2, and among them, 3 had absolute contraindications to surgery. This reflects the current state of real-world clinical practice, where elderly patients and those with multiple comorbidities often have compromised organ function. This not only increases the risks associated with the perioperative period but also means that even the choice of chemotherapy alone requires careful consideration. In this study, 5 patients were unable to complete the full course of chemotherapy due to intolerable side effects, and among them, 3 were evaluated to have SD. Nevertheless, the DCR of this study reached 100%, with a median PFS1 of 19.0 months and a 1-year PFS rate of 80%. Even patients with SD achieved a minimum PFS1 of 11 months. In this small-sample retrospective cohort, non-surgical treatment demonstrated potential signals of benefit, though validation through large-scale studies is still required.

It is widely recognized that the advent of PARP inhibitors has been a significant boon for patients with ovarian cancer. The current standard treatment paradigm for ovarian cancer includes surgery, chemotherapy (typically paclitaxel and platinum-based agents), targeted therapy (such as bevacizumab), and maintenance therapy with PARP inhibitors.^9–12^ In this study, 12 patients received PARP inhibitors (including fluzoparib and niraparib) for maintenance therapy. One patient was unable to receive PARP inhibitors due to financial constraints, while another 2 patients did not use PARP inhibitors because there were no frontline data available for the entire population before 2019.

In this study, the median PFS1 was 19.0 months. This is comparable to the median PFS of 13.8 months observed in the entire population of the PRIMA study^13^ and the median PFS of 19.6 months in the single-arm clinical study OVARIO.^14^ Notably, some patients in this study received only 3 cycles of chemotherapy and were assessed as having SD, yet they still achieved a considerably extended survival period. This extended survival may be attributed to the sequential synergistic anti-tumor effect of PARP inhibitors in combination with chemotherapy.

Cancer treatment has now entered the immunotherapy era. Combining immune checkpoint inhibitors (ICIs) with chemotherapy or targeted therapies has achieved breakthroughs in treating many solid tumors. However, progress in treating recurrent and later-line ovarian cancer with ICIs has been limited. Studies show that the response rate to ICI monotherapy in ovarian cancer patients is only 10% to 15%.^15^ ICIs work by activating T cells to fight tumors, and their effectiveness largely depends on T cell infiltration in the tumor tissue.^16^

Most ovarian cancer recurrences occur in the pelvic and abdominal regions. The omentum and pelvic lymph nodes, often removed during initial treatment, are peripheral immune organs that store mature lymphocytes and filter harmful substances, including tumor cells, from lymphatic fluid. In this study, patients received up to 6 cycles of initial chemotherapy, but about one-third completed only 3 to 4 cycles due to health reasons. Despite achieving only SD, these patients had a median PFS1 of 19 months. Could preserving the omentum and pelvic lymph nodes help maintain immune balance and control tumor progression?

Among the 10 patients with recurrence who were evaluable for PFS2, the median PFS2 was 10 months. Patients remained sensitive to chemotherapy after recurrence. For example, Patient P10 experienced 4 recurrences, using only chemotherapy and bevacizumab without PARP inhibitors, and each chemotherapy course was limited to 4 cycles, yet each PFS exceeded 6 months. The patient lived with the tumor for 57 months and ultimately died of cerebral hemorrhage, not cancer. Patient P14, an 81-year-old with multiple comorbidities (liver cancer, cirrhosis, and splenomegaly), controlled the disease for 11 months using bevacizumab and immunotherapy without chemotherapy. After progression, combining PARP inhibitors with immunotherapy controlled the disease for another 3 months. We speculate that ICIs played a significant role in this patient’s treatment.

The sample size of this study was relatively small (only 15 cases), resulting in limited statistical power; therefore, the results should be interpreted with caution. The findings of this study should be regarded as exploratory. We strongly recommend that future large-scale, prospective studies be conducted to further validate these findings.

## Conclusions

The patients in this study were either elderly or had significant comorbidities and poor physical conditions. Despite not undergoing surgery, they achieved a median OS of 57.0 months, with a 1-year OS rate of 100.0%, a 2-year OS rate of 91.7%, and a 3-year OS rate of 65.2% through comprehensive treatment. These outcomes are comparable to previously reported standard treatments.

Our study provides an alternative treatment option for elderly ovarian cancer patients. Future considerations could include using chemotherapy ± bevacizumab plus ICIs for initial treatment, followed by PARP inhibitors for maintenance therapy after disease control. We do not advocate replacing existing guidelines or making our approach the preferred method. Future prospective, multicenter clinical trials should be conducted to validate and optimize the non-surgical treatment strategy proposed in this study.

## Supplementary Material

oyaf290_Supplementary_Data

## Data Availability

The data underlying this article will be shared on reasonable request to the corresponding author.
