# Supplementary material for: Retrospective study of elderly patients with advanced ovarian cancer who did not undergo surgery
Source: Oncologist. 2025 Sep 18;30(10):oyaf290. doi: 10.1093/oncolo/oyaf290 (PMC12530882; doi:10.1093/oncolo/oyaf290)
Supplement: oyaf290_Supplementary_Data [file oyaf290_supplementary_data.docx]

Supplemental Table S1. Adverse Events

| Treatment-Emergent Adverse Events (TEAE) | Any Grade | Grade 1-2 | Grade 3 | Grade 4 |
| --- | --- | --- | --- | --- |
| Leukopenia | 14 (93.3%) | 11 (73.3%) | 2 (13.3%) | 1 (6.7%) |
| Neutropenia | 14 (93.3%) | 8 (53.3%) | 4 (26.7%) | 2 (13.3%) |
| Thrombocytopenia | 11 (73.3%) | 11 (73.3%) | —— | —— |
| Abnormal transaminases | 5 (33.3%) | 5 (33.3%) | —— | —— |
| Anemia | 13 (86.7%) | 12 (80.0%) | 1 (6.7%) | —— |
| Fatigue | 15 (100%) | 15 (100%) | —— | —— |
| Nausea | 15 (100%) | 14 (93.3%) | 1 (6.7%) | —— |
| Vomiting | 15 (100%) | 15 (100%) | —— | —— |
| Diarrhea | 6 (40.0%) | 5 (33.3%) | 1 (6.7%) | —— |

TEAE = Treatment-Emergent Adverse Events. Data are presented as number (%).
